# Bis(cyclo­hexyl­ammonium) tetra­chlorido­diphenyl­stannate(IV)

**DOI:** 10.1107/S160053681401109X

**Published:** 2014-05-17

**Authors:** Modou Sarr, Carina Merkens, Aminata Diassé-Sarr, Libasse Diop, Ulli Englert

**Affiliations:** a Laboratoire de Chimie Minerale et Analytique, Département de Chimie, Faculté des Sciences et Techniques Université Cheikh Anta Diop, Dakar, Senegal; b Institut für Anorganische Chemie, RWTH Aachen University, Landoltweg 1, 52074 Aachen, Germany

## Abstract

The title compound, (C_6_H_14_N)_2_[Sn(C_6_H_5_)_2_Cl_4_], contains cyclo­hexyl­ammonium cations in general positions and a stannate(IV) anion that is located on a twofold rotation axis. The Sn^IV^ atom in the complex anion is surrounded by four Cl^−^ ligands and two *trans*-phenyl groups in a distorted octa­hedral configuration. The anions are connected with the cations through N—H⋯Cl hydrogen bonds. Every cation is involved in three N—H⋯Cl bonds to the chloride ligands of three different anions, and each chloride ligand is linked to two cations. This arrangement leads to a layered structure parallel to (010).

## Related literature   

For applications of organotin(IV) compounds, see: Evans & Karpel (1985 ▶); Kapoor *et al.* (2005 ▶). For compounds containing the [Sn(C_6_H_5_)_2_Cl_4_]^2−^ anion in a *cis* or *trans*-conformation, see: Garcia-Seijo *et al.* (2001 ▶); Fernandez *et al.* (2002 ▶); Venkatraman *et al.* (2004 ▶); Diop *et al.* (2011 ▶). For crystal structures of related tin(IV) compounds, see: Sarr *et al.* (2013*a* ▶,*b* ▶).
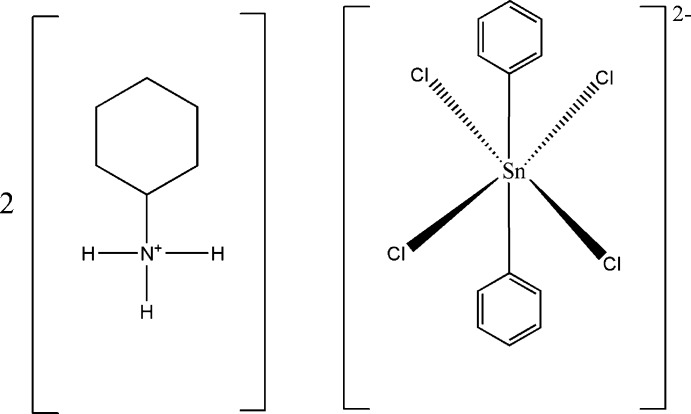


## Experimental   

### 

#### Crystal data  

(C_6_H_14_N)_2_[Sn(C_6_H_5_)_2_Cl_4_]
*M* *_r_* = 615.05
Orthorhombic, 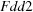

*a* = 13.558 (4) Å
*b* = 49.646 (14) Å
*c* = 8.058 (2) Å
*V* = 5424 (3) Å^3^
*Z* = 8
Mo *K*α radiation
μ = 1.35 mm^−1^
*T* = 100 K
0.30 × 0.21 × 0.05 mm

#### Data collection  

Bruker D8 goniometer with APEX area detector
Absorption correction: multi-scan (*SADABS*; Bruker, 2009 ▶) *T*_min_ = 0.687, *T*_max_ = 0.935
15474 measured reflections
2772 independent reflections
2563 reflections with *I* > 2σ(*I*)
*R*_int_ = 0.069

#### Refinement  

*R*[*F* ^2^ > 2σ(*F* ^2^)] = 0.043
*wR*(*F* ^2^) = 0.100
*S* = 1.06
2772 reflections
151 parameters
4 restraints
H atoms treated by a mixture of independent and constrained refinement
Δρ_max_ = 1.92 e Å^−3^
Δρ_min_ = −0.67 e Å^−3^
Absolute structure: Flack (1983 ▶), 1281 Friedel pairs
Absolute structure parameter: 0.23 (5)

### 

Data collection: *SMART* (Bruker, 2009 ▶); cell refinement: *SAINT* (Bruker, 2009 ▶); data reduction: *SAINT*; program(s) used to solve structure: *SHELXS97* (Sheldrick, 2008 ▶); program(s) used to refine structure: *SHELXL2013* (Sheldrick, 2008 ▶); molecular graphics: *PLATON* (Spek, 2009 ▶); software used to prepare material for publication: *SHELXL2013*.

## Supplementary Material

Crystal structure: contains datablock(s) global, I. DOI: 10.1107/S160053681401109X/wm5023sup1.cif

Structure factors: contains datablock(s) I. DOI: 10.1107/S160053681401109X/wm5023Isup2.hkl

CCDC reference: 1002989

Additional supporting information:  crystallographic information; 3D view; checkCIF report

## Figures and Tables

**Table 1:** Hydrogen-bond geometry (Å, °)

*D*—H⋯*A*	*D*—H	H⋯*A*	*D*⋯*A*	*D*—H⋯*A*
N1—H1*A*⋯Cl2^i^	0.91 (3)	2.35 (4)	3.244 (8)	166 (10)
N1—H1*B*⋯Cl1^ii^	0.91 (3)	2.36 (6)	3.172 (9)	148 (8)
N1—H1*C*⋯Cl2^iii^	0.90 (3)	2.60 (7)	3.328 (9)	139 (9)

Symmetry codes: (i) 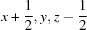; (ii) 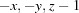; (iii) 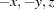.

**Fig. 1.**
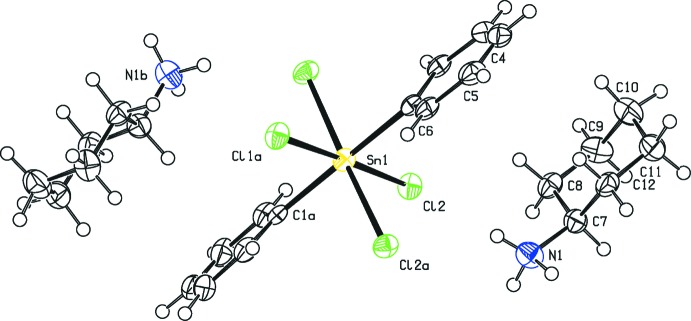
The molecular entities of the title compound with partial atom labelling. [Symmetry code: (i) -x, -y, z.]

**Fig. 2.**
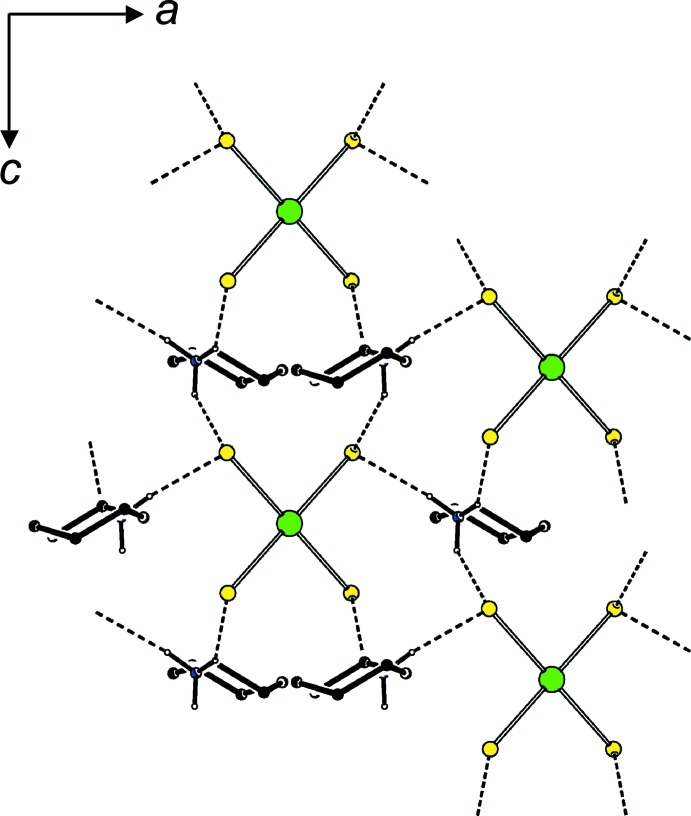
A view of N—H···Cl hydrogen bonds in the crystal structure of the title compound. H atoms non-participating in hydrogen bonding and the phenyl groups have been omitted for clarity.

## supplementary crystallographic information

### 1. Comment

Our interest for organotin(IV) compounds (Sarr *et al.*, 2013**a*,b*) is related to applications found in various fields like in medicine, industry or agriculture (Evans & Karpel, 1985; Kapoor *et al.*, 2005).

The crystal structure of the title compound, 2(C_6_H_14_N)^+^[Sn(C_6_H_5_)_2_Cl_4_]^2-^, consists of cyclohexylammonium cations and a [SnPh_2_Cl_4_]^2-^ anion that is located on a twofold rotation axis. The Sn^IV^ atom is bonded to two *trans*-phenyl groups and four chloride ligands in a distorted octahedral geometry (Fig. 1). For reasons of symmetry, the Sn—C bond lengths are equal and amount to 2.142 (5) Å. The two independent Sn—Cl bond lengths have very similar values [2.5685 (16) and 2.5842 (17) Å] and may be compared to the values of 2.5722 (6) and 2.5796 (6) Å reported for bis(trimethylammonium) tetrachloridodiphenylstannate(IV) (Diop *et al.*, 2011). The C—Sn—C angle (179.6 (4) °) is linear within experimental error. The angles in the equatorial plane of the pseudo-octahedron deviate slightly from 90° [Cl1—Sn1—Cl1^i^ = 91.12 (8)°; Cl1—Sn1—Cl2 = 89.41 (5)°, i = -*x*, -*y*, *z*]. The tetrachloridodiphenylstannate(IV anion, [SnPh_2_Cl_4_]^2-^, in its *cis* or *trans* configurations has been reported previously by several authors with different counter cations (Garcia-Seijo *et al.*, 2001; Fernandez *et al.*, 2002; Venkatraman *et al.*, 2004; Diop *et al.*, 2011).

Each cation in the title compound is linked to Cl atoms of three different anions through classical N—H···Cl hydrogen bonds (Fig. 2, Table 1), leading to a layered arrangement parallel to (010).

### 2. Experimental

Equimolar amounts of cyclohexylamin and oxalic were dissolved in water; crystals formed by slow evaporation. Their elemental analyses, calculated/ % (found/ %), C: 53.31 (53.05); %H: 9.91(10.28); %N: 8.88(8.40), suggest the composition (CyNH_3_)_2_(C_2_O_4_)^.^3/2H_2_O. Crystals suitable for the X-ray determination of the title compound were obtained by mixing methanolic solutions of (CyNH_3_)_2_(C_2_O_4_)^.^3/2H_2_O and SnPh_2_Cl_2_ in a 1:1 ratio and subsequent slow solvent evaporation.

### 3. Refinement

Hydrogen atoms bonded to carbon were treated as riding with C–H = 0.95 Å for aryl-CH and C–H = 0.99 Å for CH_2_ groups. Isotropic displacement parameters for these hydrogen atoms were constrained to *U*_iso_(H) = 1.2*U*_eq_(C). Hydrogen atoms bonded to nitrogen were located in a difference Fourier map; N—H distances were restrained to 0.91 (3) Å. Isotropic displacement parameters for these hydrogen atoms were constrained to *U*_iso_(H) = 1.5*U*_eq_(N). Refinement showed that the crystal under investigation was an inversion twin; the major domain is associated with a volume fraction of 0.77 (5).

### Crystal data

**Table:**

(C_6_H_14_N)_2_[Sn(C_6_H_5_)_2_Cl_4_]	*F*(000) = 2512
*M**_r_* = 615.05	*D*_x_ = 1.506 Mg m^−^^3^
Orthorhombic, *F**d**d*2	Mo *K*α radiation, λ = 0.71073 Å
Hall symbol: F 2 -2d	Cell parameters from 5810 reflections
*a* = 13.558 (4) Å	θ = 3.0–26.2°
*b* = 49.646 (14) Å	µ = 1.35 mm^−^^1^
*c* = 8.058 (2) Å	*T* = 100 K
*V* = 5424 (3) Å^3^	Plate, colorless
*Z* = 8	0.30 × 0.21 × 0.05 mm

### Data collection

**Table:**

Bruker D8 goniometer with APEX area detector diffractometer	2772 independent reflections
Radiation source: Incoatec microsource	2563 reflections with *I* > 2σ(*I*)
Multilayer optics monochromator	*R*_int_ = 0.069
ω scans	θ_max_ = 26.5°, θ_min_ = 3.0°
Absorption correction: multi-scan (*SADABS*; Bruker, 2009)	*h* = −16→16
*T*_min_ = 0.687, *T*_max_ = 0.935	*k* = −62→62
15474 measured reflections	*l* = −10→10

### Refinement

**Table:**

Refinement on *F*^2^	Secondary atom site location: difference Fourier map
Least-squares matrix: full	Hydrogen site location: inferred from neighbouring sites
*R*[*F*^2^ > 2σ(*F*^2^)] = 0.043	H atoms treated by a mixture of independent and constrained refinement
*wR*(*F*^2^) = 0.100	*w* = 1/[σ^2^(*F*_o_^2^) + (0.020*P*)^2^] where *P* = (*F*_o_^2^ + 2*F*_c_^2^)/3
*S* = 1.06	(Δ/σ)_max_ = 0.004
2772 reflections	Δρ_max_ = 1.92 e Å^−^^3^
151 parameters	Δρ_min_ = −0.67 e Å^−^^3^
4 restraints	Absolute structure: Flack (1983), 1281 Friedel pairs
Primary atom site location: structure-invariant direct methods	Absolute structure parameter: 0.23 (5)

### Special details

**Table:**

Geometry. All e.s.d.'s (except the e.s.d. in the dihedral angle between two l.s. planes) are estimated using the full covariance matrix. The cell e.s.d.'s are taken into account individually in the estimation of e.s.d.'s in distances, angles and torsion angles; correlations between e.s.d.'s in cell parameters are only used when they are defined by crystal symmetry. An approximate (isotropic) treatment of cell e.s.d.'s is used for estimating e.s.d.'s involving l.s. planes.
Refinement. Refinement of *F*^2^ against ALL reflections. The weighted *R*-factor *wR* and goodness of fit *S* are based on *F*^2^, conventional *R*-factors *R* are based on *F*, with *F* set to zero for negative *F*^2^. The threshold expression of *F*^2^ > σ(*F*^2^) is used only for calculating *R*-factors(gt) *etc*. and is not relevant to the choice of reflections for refinement. *R*-factors based on *F*^2^ are statistically about twice as large as those based on *F*, and *R*- factors based on ALL data will be even larger.

### Fractional atomic coordinates and isotropic or equivalent isotropic displacement parameters (Å^2^)

**Table:**

	*x*	*y*	*z*	*U*_iso_*/*U*_eq_	
Sn1	−0.0000	−0.0000	0.72474 (9)	0.02578 (15)	
Cl1	−0.11741 (11)	0.01834 (3)	0.94793 (18)	0.0329 (4)	
Cl2	−0.11954 (10)	0.01707 (3)	0.4982 (2)	0.0308 (4)	
C1	0.0727 (4)	0.03830 (10)	0.7238 (8)	0.0285 (11)	
C2	0.0212 (3)	0.06170 (10)	0.7321 (11)	0.0277 (12)	
H2	−0.0484	0.0609	0.7439	0.033*	
C3	0.0659 (4)	0.08670 (11)	0.7241 (9)	0.0362 (13)	
H3	0.0275	0.1027	0.7226	0.043*	
C4	0.1690 (4)	0.08788 (11)	0.7183 (9)	0.0357 (13)	
H4	0.2016	0.1048	0.7182	0.043*	
C5	0.2215 (5)	0.06497 (13)	0.7128 (10)	0.0372 (14)	
H5	0.2914	0.0659	0.7060	0.045*	
C6	0.1752 (4)	0.03965 (11)	0.7170 (9)	0.0297 (12)	
H6	0.2134	0.0236	0.7152	0.036*	
N1	0.1784 (4)	0.02720 (11)	0.2043 (9)	0.0375 (13)	
H1A	0.233 (4)	0.0271 (15)	0.138 (8)	0.056*	
H1B	0.142 (5)	0.0132 (10)	0.164 (8)	0.056*	
H1C	0.181 (6)	0.0222 (15)	0.311 (4)	0.056*	
C7	0.1431 (4)	0.05466 (11)	0.1699 (7)	0.0317 (14)	
H7	0.1271	0.0557	0.0490	0.038*	
C8	0.0488 (4)	0.06018 (12)	0.2660 (7)	0.0326 (15)	
H8A	−0.0029	0.0472	0.2316	0.039*	
H8B	0.0610	0.0577	0.3862	0.039*	
C9	0.0141 (4)	0.08851 (11)	0.2336 (16)	0.0401 (14)	
H9A	−0.0452	0.0921	0.3018	0.048*	
H9B	−0.0050	0.0902	0.1155	0.048*	
C10	0.0917 (5)	0.10933 (13)	0.2730 (9)	0.0489 (19)	
H10A	0.1054	0.1093	0.3937	0.059*	
H10B	0.0674	0.1274	0.2419	0.059*	
C11	0.1854 (5)	0.10318 (12)	0.1787 (8)	0.0417 (17)	
H11A	0.1735	0.1060	0.0588	0.050*	
H11B	0.2374	0.1160	0.2138	0.050*	
C12	0.2216 (5)	0.07509 (12)	0.2055 (10)	0.0343 (14)	
H12A	0.2438	0.0731	0.3219	0.041*	
H12B	0.2790	0.0717	0.1325	0.041*	

### Atomic displacement parameters (Å^2^)

**Table:**

	*U*^11^	*U*^22^	*U*^33^	*U*^12^	*U*^13^	*U*^23^
Sn1	0.0195 (2)	0.0327 (3)	0.0251 (2)	−0.0025 (2)	−0.000	−0.000
Cl1	0.0289 (7)	0.0418 (8)	0.0281 (11)	−0.0032 (6)	0.0082 (7)	−0.0044 (7)
Cl2	0.0236 (6)	0.0372 (7)	0.0316 (11)	−0.0016 (5)	−0.0062 (7)	0.0032 (7)
C1	0.040 (3)	0.031 (3)	0.014 (2)	−0.002 (2)	−0.006 (3)	0.001 (3)
C2	0.020 (3)	0.042 (3)	0.021 (3)	0.000 (2)	0.009 (4)	−0.002 (3)
C3	0.048 (4)	0.033 (3)	0.028 (3)	0.002 (2)	0.002 (4)	−0.006 (3)
C4	0.045 (3)	0.037 (3)	0.025 (3)	−0.013 (3)	−0.006 (3)	−0.002 (3)
C5	0.037 (3)	0.047 (4)	0.028 (3)	−0.012 (3)	0.001 (3)	−0.007 (4)
C6	0.025 (3)	0.041 (3)	0.023 (3)	−0.001 (2)	0.010 (3)	−0.002 (3)
N1	0.030 (3)	0.035 (3)	0.047 (4)	0.002 (2)	0.005 (3)	−0.003 (3)
C7	0.031 (3)	0.031 (3)	0.032 (3)	−0.001 (3)	0.003 (2)	−0.000 (2)
C8	0.028 (3)	0.043 (4)	0.027 (4)	−0.004 (3)	−0.001 (2)	0.001 (2)
C9	0.027 (3)	0.041 (3)	0.052 (4)	0.008 (3)	−0.002 (4)	−0.009 (5)
C10	0.060 (4)	0.032 (3)	0.056 (5)	0.005 (3)	0.003 (3)	−0.002 (3)
C11	0.047 (4)	0.037 (4)	0.041 (4)	−0.010 (3)	−0.000 (3)	−0.001 (3)
C12	0.036 (3)	0.041 (3)	0.026 (4)	−0.011 (3)	0.005 (3)	−0.001 (3)

### Geometric parameters (Å, º)

**Table:**

Sn1—C1^i^	2.142 (5)	N1—H1B	0.91 (2)
Sn1—C1	2.142 (5)	N1—H1C	0.90 (2)
Sn1—Cl1	2.5685 (16)	C7—C12	1.498 (8)
Sn1—Cl1^i^	2.5686 (16)	C7—C8	1.519 (8)
Sn1—Cl2	2.5842 (17)	C7—H7	1.0000
Sn1—Cl2^i^	2.5843 (17)	C8—C9	1.506 (8)
C1—C2	1.357 (7)	C8—H8A	0.9900
C1—C6	1.392 (7)	C8—H8B	0.9900
C2—C3	1.383 (7)	C9—C10	1.509 (9)
C2—H2	0.9500	C9—H9A	0.9900
C3—C4	1.399 (8)	C9—H9B	0.9900
C3—H3	0.9500	C10—C11	1.511 (9)
C4—C5	1.343 (9)	C10—H10A	0.9900
C4—H4	0.9500	C10—H10B	0.9900
C5—C6	1.406 (8)	C11—C12	1.494 (9)
C5—H5	0.9500	C11—H11A	0.9900
C6—H6	0.9500	C11—H11B	0.9900
N1—C7	1.471 (8)	C12—H12A	0.9900
N1—H1A	0.91 (2)	C12—H12B	0.9900
			
C1^i^—Sn1—C1	179.6 (4)	H1B—N1—H1C	99 (7)
C1^i^—Sn1—Cl1	91.82 (16)	N1—C7—C12	111.1 (5)
C1—Sn1—Cl1	88.46 (17)	N1—C7—C8	110.2 (5)
C1^i^—Sn1—Cl1^i^	88.45 (17)	C12—C7—C8	112.2 (5)
C1—Sn1—Cl1^i^	91.82 (16)	N1—C7—H7	107.7
Cl1—Sn1—Cl1^i^	91.12 (8)	C12—C7—H7	107.7
C1^i^—Sn1—Cl2	90.00 (17)	C8—C7—H7	107.7
C1—Sn1—Cl2	89.72 (16)	C9—C8—C7	110.1 (6)
Cl1—Sn1—Cl2	89.41 (5)	C9—C8—H8A	109.6
Cl1^i^—Sn1—Cl2	178.38 (5)	C7—C8—H8A	109.6
C1^i^—Sn1—Cl2^i^	89.72 (16)	C9—C8—H8B	109.6
C1—Sn1—Cl2^i^	90.00 (17)	C7—C8—H8B	109.6
Cl1—Sn1—Cl2^i^	178.39 (5)	H8A—C8—H8B	108.2
Cl1^i^—Sn1—Cl2^i^	89.41 (5)	C8—C9—C10	112.6 (6)
Cl2—Sn1—Cl2^i^	90.10 (8)	C8—C9—H9A	109.1
C2—C1—C6	118.3 (5)	C10—C9—H9A	109.1
C2—C1—Sn1	121.5 (4)	C8—C9—H9B	109.1
C6—C1—Sn1	120.1 (4)	C10—C9—H9B	109.1
C1—C2—C3	122.7 (5)	H9A—C9—H9B	107.8
C1—C2—H2	118.6	C9—C10—C11	110.0 (6)
C3—C2—H2	118.6	C9—C10—H10A	109.7
C2—C3—C4	118.5 (5)	C11—C10—H10A	109.7
C2—C3—H3	120.8	C9—C10—H10B	109.7
C4—C3—H3	120.8	C11—C10—H10B	109.7
C5—C4—C3	119.7 (5)	H10A—C10—H10B	108.2
C5—C4—H4	120.2	C12—C11—C10	113.1 (5)
C3—C4—H4	120.2	C12—C11—H11A	109.0
C4—C5—C6	121.3 (6)	C10—C11—H11A	109.0
C4—C5—H5	119.3	C12—C11—H11B	109.0
C6—C5—H5	119.3	C10—C11—H11B	109.0
C1—C6—C5	119.3 (5)	H11A—C11—H11B	107.8
C1—C6—H6	120.3	C11—C12—C7	111.8 (5)
C5—C6—H6	120.3	C11—C12—H12A	109.3
C7—N1—H1A	99 (5)	C7—C12—H12A	109.3
C7—N1—H1B	118 (5)	C11—C12—H12B	109.3
H1A—N1—H1B	103 (6)	C7—C12—H12B	109.3
C7—N1—H1C	117 (5)	H12A—C12—H12B	107.9
H1A—N1—H1C	122 (7)		
			
C1^i^—Sn1—C1—C2	93.3 (8)	C2—C3—C4—C5	3.1 (11)
Cl1—Sn1—C1—C2	−41.0 (6)	C3—C4—C5—C6	−1.6 (12)
Cl1^i^—Sn1—C1—C2	−132.1 (6)	C2—C1—C6—C5	−2.3 (10)
Cl2—Sn1—C1—C2	48.4 (6)	Sn1—C1—C6—C5	178.6 (6)
Cl2^i^—Sn1—C1—C2	138.5 (6)	C4—C5—C6—C1	1.2 (11)
C1^i^—Sn1—C1—C6	−87.6 (4)	N1—C7—C8—C9	178.7 (6)
Cl1—Sn1—C1—C6	138.0 (5)	C12—C7—C8—C9	54.3 (7)
Cl1^i^—Sn1—C1—C6	47.0 (5)	C7—C8—C9—C10	−55.7 (10)
Cl2—Sn1—C1—C6	−132.5 (5)	C8—C9—C10—C11	55.1 (10)
Cl2^i^—Sn1—C1—C6	−42.4 (5)	C9—C10—C11—C12	−53.5 (8)
C6—C1—C2—C3	4.0 (12)	C10—C11—C12—C7	53.4 (8)
Sn1—C1—C2—C3	−177.0 (6)	N1—C7—C12—C11	−177.3 (6)
C1—C2—C3—C4	−4.4 (12)	C8—C7—C12—C11	−53.5 (7)

Symmetry code: (i) −*x*, −*y*, *z*.

### Hydrogen-bond geometry (Å, º)

**Table:**

*D*—H···*A*	*D*—H	H···*A*	*D*···*A*	*D*—H···*A*
N1—H1*A*···Cl2^ii^	0.91 (3)	2.35 (4)	3.244 (8)	166 (10)
N1—H1*B*···Cl1^iii^	0.91 (3)	2.36 (6)	3.172 (9)	148 (8)
N1—H1*C*···Cl2^i^	0.90 (3)	2.60 (7)	3.328 (9)	139 (9)

Symmetry codes: (i) −*x*, −*y*, *z*; (ii) *x*+1/2, *y*, *z*−1/2; (iii) −*x*, −*y*, *z*−1.
